# Post-transplant cyclophosphamide after matched sibling, unrelated and haploidentical donor transplants in patients with acute myeloid leukemia: a comparative study of the ALWP EBMT

**DOI:** 10.1186/s13045-020-00882-6

**Published:** 2020-05-06

**Authors:** Jaime Sanz, Jacques-Emmanuel Galimard, Myriam Labopin, Boris Afanasyev, Emanuele Angelucci, Fabio Ciceri, Didier Blaise, Jan J. Cornelissen, Ellen Meijer, J. L. Diez-Martin, Yener Koc, Montserrat Rovira, Luca Castagna, Bipin Savani, Annalisa Ruggeri, Arnon Nagler, Mohamad Mohty, Acute Leukemia Working Party of the European Society for Blood and Marrow Transplantation (EBMT)

**Affiliations:** 1 Hematology Department, Hospital Universitari i Politècnic La Fe, Avinguda Fernando Abril Martorell, 106, 46026 Valencia, Spain; 2 CIBERONC, Instituto Carlos III, Madrid, Spain; 3 EBMT Paris Office, Hospital Saint Antoine, Paris, France; 4 Department of Hematology, Hopital Saint Antoine, Sorbonne University, Paris, France; 5 First State Pavlov Medical University of St. Petersburg, Raisa Gorbacheva Memorial Research Institute for Paediatric Oncology, Hematology, and Transplantation, St Petersburg, Russia; 6 Department of Haematology, IRCCS Ospedale Policlinico San Martino, Genova, Italy; 7 Haematology and BMT, Ospedale San Raffaele s.r.l., Milano, Italy; 8 Programme de Transplantation & Therapie Cellulaire, Centre de Recherche en Cancérologie de Marseille, Institut Paoli Calmettes, Marseille, France; 9 Department of Hematology, Erasmus MC Cancer Institute, University Medical Center Rotterdam, Rotterdam, The Netherlands; 10 Department of Hematology (Br 250), VU University Medical Center, Amsterdam, The Netherlands; 11 Hematology Department, Hospital GU Gregorio Marañon, Instituto de Investigación sanitaria Gregorio Marañon, Universidad Complutense Madrid, Madrid, Spain; 12 Stem Cell Transplant Unit, Medical Park Hospitals, Antalya, Turkey; 13 Dept. of Hematology, Institute of Hematology & Oncology, Hospital Clinic, Barcelona, Spain; 14 Institut d’Investigació Biomèdica August Pi I Sunyer (IDIBAPS), Institut Josep Carreras, University of Barcelona, Barcelona, Spain; 15 Transplantation Unit, Department of Oncology and Haematology, Istituto Clinico Humanitas, Milan, Italy; 16 Vanderbilt University Medical Center, Nashville, TN USA; 17 Department of Pediatric Hematology and Oncology, IRCCS Bambino Gesù Children’s Hospital, Piazza S.Onofrio, 4, Rome, Italy; 18 Division of Hematology and Bone Marrow Transplantation, The Chaim Sheba Medical Center, Tel-Hashomer, Ramat-Gan, Israel; 19 ALWP of the EBMT office, Saint Antoine Hospital, Paris, France

**Keywords:** Post-transplant cyclophosphamide, Haploidentical transplant, Alternative donor transplants, Acute leukemia, Allogeneic stem cell transplant

## Abstract

**Background:**

The use of post-transplant cyclophosphamide (PTCy) is highly effective in preventing graft-versus-host disease (GVHD) in the haploidentical (Haplo) transplant setting and is being increasingly used in matched sibling (MSD) and matched unrelated (MUD) transplants. There is no information on the impact of donor types using homogeneous prophylaxis with PTCy.

**Methods:**

We retrospectively compared outcomes of adult patients with acute myeloid leukemia (AML) in first complete remission (CR1) who received a first allogeneic stem cell transplantation (SCT) with PTCy as GVHD prophylaxis from MSD (*n* = 215), MUD (*n* = 235), and Haplo (*n* = 789) donors registered in the EBMT database between 2010 and 2017.

**Results:**

The median follow-up was 2 years. Haplo-SCT carried a significantly increased risk of acute grade II–IV GVHD (HR 1.6; 95% CI 1.1–2.4) and NRM (HR 2.6; 95% CI 1.5–4.5) but a lower risk of relapse (HR 0.7; 95% CI 0.5–0.9) that translated to no differences in LFS (HR 1.1; 95% CI 0.8–1.4) or GVHD/relapse-free survival (HR 1; 95% CI 0.8–1.3). Interestingly, the use of peripheral blood was associated with an increased risk of acute (HR 1.9; 95% CI 1.4–2.6) and chronic GVHD (HR 1.7; 95% CI 1.2–2.4) but a lower risk of relapse (HR 0.7; 95% CI 0.5–0.9).

**Conclusions:**

The use of PTCy in patients with AML in CR1 receiving SCT from MSD, MUD, and Haplo is safe and effective. Haplo-SCT had increased risk of acute GVHD and NRM and lower relapse incidence but no significant difference in survival.

## Introduction

The use of post-transplant cyclophosphamide (PTCy) has proven to be highly effective in preventing graft-versus-host (GVHD) and reducing non-relapse mortality (NRM) rates in haploidentical (Haplo) hematopoietic stem cell transplant (SCT) [1–3].

As a consequence, PTCy is being increasingly used in other allogeneic transplant settings, such as SCT from HLA-matched sibling donors (MSD) [4–6], matched unrelated donors (MUD), and mismatched unrelated donors (MMUD) [1–3, 7].

Since the first reports of Haplo-SCT using PTCy, there has been significant interest in comparing this platform with those using other donor types such as cord blood from unrelated donors [4–6, 8] or bone marrow (BM) or peripheral blood (PB) from MSD, MUD, and MMUD transplants [9–15]. However, an important limitation of these studies is that each type of transplant received different GVHD prophylaxis. Therefore, comparisons were made of different transplant platforms instead of different donor types. Two prospective studies compared T cell replete Haplo and MSD transplants in acute myeloid leukemia (AML) [16] and acute lymphoblastic leukemia [17] using non-PTCy GVHD prophylaxis with similar results but increased risk of GVHD after Haplo. In addition, safety and feasibility of PTCy-based GVHD prophylaxis in transplants using different donor types was further evaluated in small single-center prospective non-randomized studies showing comparable outcomes in Haplo-SCT compared to 9/10 MUD [18], as well as in MSD, MUD, and MMUD transplants [19].

The aim of this study was to investigate the impact of donor type in the outcome of patients with AML undergoing unmanipulated allogeneic SCT (allo-SCT) using PTCy as GvHD prophylaxis. We analyzed patients who received allo-SCT from MSD, MUD, and Haplo for acute myeloid leukemia (AML) in first complete remission (CR1) and reported to the European Society for Blood and Marrow Transplantation (EBMT) registry from 2010 to 2017.

## Patients and methods

### Study design and data source

This is a retrospective registry-based analysis on behalf of the Acute Leukaemia Working Party (ALWP) of the EBMT. The EBMT is a voluntary working group of more than 600 transplantation centers that are required to report all consecutive stem cell transplantations and follow-up once a year. Audits are routinely performed to determine the accuracy of the data. All transplantation centers are required to obtain written informed consent before data registration with the EBMT in accordance with the 1975 Helsinki Declaration.

### Patient eligibility

All adults (age ≥ 18 years) with AML in CR1 at transplantation, reported to Promise-EBMT, who underwent first allogeneic SCT, from an unmanipulated graft, using PT-Cy from Haplo, MUD, or MSD donors between 2010 and 2017, were analyzed. Haplo was defined as recipient-donor number of human leukocyte antigen (HLA) mismatches ≥ 2.

### Endpoints and definitions

The primary endpoint was to compare leukemia-free survival (LFS) after MSD, MUD, and Haplo donor transplants. Secondary endpoints were neutrophil engraftment, acute GVHD (aGVHD) and chronic GVHD (cGVHD), relapse incidence, nonrelapse mortality (NRM), GVHD-free and relapse-free survival (GRFS), and overall survival (OS) within the same subgroups and to perform analysis of risk factors for each outcome.

Neutrophil recovery was defined as the first day of an absolute neutrophil count of 0.5 × 10^9^/L lasting for 3 or more consecutive days. aGVHD and cGVHD were defined and graded according to standard criteria [20, 21]. Relapse was defined as disease recurrence and appearance of blasts in the peripheral blood or BM (> 5%) after CR. LFS was calculated until the date of first relapse, death from any cause, or the last follow-up for patients in CR. NRM was defined as death from any cause other than relapse. The composite endpoint GRFS was defined as survival without the following events: stage III–IV aGVHD, severe cGVHD, disease relapse, or death from any cause after SCT [22]. Myeloablative conditioning (MAC) was defined as a regimen containing either total body irradiation with a dose greater than 6 Gray, a total dose of oral busulfan greater than 8 mg/kg, or a total dose of intravenous busulfan > 6.4 mg/kg or melphalan at doses > 140 mg/m^2^. In addition, regimens containing 2 alkylating agents were considered as MAC. All other regimens were defined as reduced intensity (RIC).

### Statistical analysis

Patient characteristics according to donor type were compared using chi-squared tests for categorical and Kruskal-Wallis tests for continuous variables. GRFS, LFS, and OS were estimated using the Kaplan-Meier method. Cumulative incidence functions were used to estimate neutrophil engraftment, aGVHD, cGVHD, relapse incidence, and NRM. Competing risks were death for relapse incidence and neutrophil engraftment, relapse for NRM, and relapse or death for aGVHD and cGVHD. Univariate analyses were done using the log-rank test for LFS, GRFS, and OS and Gray’s test for cumulative incidence. Multivariate analyses were performed using the Cox proportional hazard model.

Donor type, gender, age at transplantation, performance status, cytogenetic risk group according to the Medical Research Council [23], type of AML (primary vs secondary) stem cell source, transplantation year, cytomegalovirus serostatus, and conditioning regimen were included in the final model. To take into account the center effect, we introduced a random effect (also named frailty effect) for each center into the model. The significance level was fixed at .05, and *p* values were 2-sided. Statistical analyses were performed using R software version 3.2.3 (R Development Core Team, Vienna, Austria) software packages.

## Results

### Patient and transplantation characteristics

Patient, disease, and transplant characteristics of the overall population and according to donor type are summarized in Table 1. Briefly, a total of 1239 patients were included in the study, of which 789 were transplanted from Haplo, 235 from MUD, and 215 from MSD donors. Median age of patients was 52 years (range, 18–76). Forty-seven (6%), 543 (66%), and 239 (29%) had standard-, intermediate-, and high-risk cytogenetics, respectively. Preferred conditioning regimens were thiotepa, busulfan, and fludarabine for Haplo (*n* = 371; 47%) and busulfan and fludarabine for MSD (*n* = 83; 39%) and MUD (*n* = 102; 43%).

**Table 1:** Patient disease and transplant characteristics according to donor type

Characteristics	Total *N* = 1239	MSD *N* = 215	MUD *N* = 235	Haplo *N* = 789	*p*
Age in years, median (range)	52 (18–75)	48 (18–71)	47 (18–74)	54 (18–75)	< 0.001
Gender, *n* (%)					0.9
Male	693 (56)	117 (54)	132 (56)	444 (56)	
Female	546 (44)	98 (46)	103 (44)	345 (44)	
Karnofsky performance status, *n* (%)					0.2
≥ 90	929 (79)	159 (77)	192 (83)	578 (78)	
< 90	251 (21)	48 (23)	39 (17)	164 (22)	
Missing	59	8	4	47	
Cytogenetic risk category, *n* (%)					0.2
Standard	47 (6)	10 (8)	8 (6)	29 (5)	
Intermediate	543 (66)	92 (70)	87 (60)	364 (66)	
High	239 (29)	29 (22)	49 (34)	161 (29)	
Missing	410	84	91	235	
Type of AML, *n* (%)					< 0.001
De novo	1046 (84)	188 (87)	216 (92)	642 (81)	
Secondary	193 (16)	27 (13)	19 (8)	147 (19)	
Months from diagnosis to transplant, median (range)	5 (1-18)	4 (1-18)	5 (2-18)	5 (1-18)	< 0.001
Conditioning intensity, *n* (%)					0.03
Myeloablative	725 (59)	122 (58)	116 (50)	487 (62)	
Reduced intensity	500 (41)	87 (42)	115 (50)	298 (38)	
Missing	14	6	4	4	
Type of conditioning, *n* (%)					0.2
Based on chemotherapy	950 (77)	159 (75)	172 (75)	619 (78)	
Based on TBI	287 (23)	54 (25)	63 (25)	170 (22)	
Missing	2	2	0	0	
Stem cell source, *n* (%)					< 0.001
Bone marrow	425 (34)	62 (29)	22 (9)	341 (43)	
Mobilized peripheral blood	814 (66)	152 (71)	213 (91)	448 (57)	
In vivo T cell depletion, *n* (%)	164 (13)	29 (13)	63 (27)	72 (9)	< 0.001
GvHD prophylaxis, *n* (%)					< 0.001
PTCy + 2 drugs	897 (72)	56 (26)	111 (47)	730 (93)	
PTCy + 1 drug	265 (21)	108 (50)	111 (47)	46 (6)	
PTCy only	77 (6)	51 (24)	13 (6)	13 (2)	
Donor-recipient gender combination, *n* (%)					0.02
Female donor to male recipient	249 (20)	54 (25)	34 (14)	161 (20)	
Other combinations	988 (80)	161 (75)	201 (86)	626 (80)	
Missing	2	0	0	2	
Donor-recipient CMV serostatus, *n* (%)					< 0.001
Negative-negative	181 (15)	33 (16)	49 (21)	99 (13)	
Positive-negative	82 (7)	13 (6)	13 (6)	56 (7)	
Negative-positive	248 (21)	17 (13)	73 (32)	148 (19)	
Positive-positive	680 (57)	129 (64)	94 (41)	457 (60)	
Missing	48	13	6	29	

*TBI* total-body irradiation, *CMV* cytomegalovirus

Haplo patients were older (*p* < 0.001) and had higher proportion of secondary AML (*p* < 0.001), while differences in gender, performance status, and cytogenetic risk category were not statistically significant. Regarding transplant characteristics, Haplo-SCT recipients received more frequently MAC (*p* = 0.006) and BM as stem cell source (*p* < 0.001), while the proportion of in vivo T cell depletion was higher in MUD transplants (*p* < 0.001). Although the vast majority (93%) of Haplo patients received GvHD prophylaxis with PTCy combined with 2 other immunosuppressive (IS) drugs, only 47% and 26% of MUD and MSD patients received such combination, respectively. In contrast, a higher proportion of MSD and MUD patients received 1 or no additional IS drugs than Haplo patients (*p* < 0.001).

### Engraftment

Cumulative incidence of neutrophil recovery at 60 days was 94% (95% CI; 92–95) for Haplo, 98% (95% CI; 95–99) for MUDs, and 98% (95% CI; 94–99) for MSDs (*p* = 0.12). The median time of neutrophil recovery was 19 days (range, 2–63), 20 days (range, 2–48), and 19 days (range, 5–64) for Haplo, MUD, and MSD, respectively.

### GvHD

The cumulative incidence of aGvHD grades II–IV at 100 days was 26% (95% CI; 23–29) for Haplo, 28% (95% CI; 22–34) for MUD, and 17% (95% CI; 12–23) for MSD (*p* = 0.03). The cumulative incidence of aGvHD grades III–IV was 9% (95% CI; 7–12) for Haplo, 8% (95% CI; 5–11) for MUD, and 6% (95% CI; 4–10) for MSD (*p* = 0.2) (Table 2). In multivariable analysis (Table 3), Haplo was associated with an increased risk of aGvHD grades II–IV, when compared with MSD (HR 1.6; 95% CI, 1.08–2.37; *p* = 0.02). The use of PB as the stem cell source was also associated with a higher risk of aGvHD grades II–IV (HR 1.93; 95% CI, 1.39–2.67; *p* < 0.001) and grades III–IV (HR 1.86; 95% CI, 1.1–3.15; *p* = 0.02) (Table 4).

**Table 2:** Univariable analysis of transplants outcomes according to donor type

Outcome*	MSD	MUD	Haplo	*p*
Acute GvHD, % (95% CI)				
Grades II–IV	17 (12–23)	28 (22–34)	26 (23–29)	0.03
Grades III–IV	6 (4–10)	8 (5–11)	9 (7–12)	0.2
Chronic GvHD, % (95% CI)				
Overall	34 (26–41)	32 (25–39)	30 (26–33)	0.3
Extensive type	14 (9–20)	18 (13–25)	10 (8–13)	0.003
NRM, % (95% CI)	10 (6–15))	14 (9–19)	23 (20–26)	< 0.001
RI, % (95% CI)	33 (26–40)	25 (19–31)	23 (20–26)	0.02
LFS, % (95% CI)	57 (49–65)	62 (55–69)	54 (51–58)	0.2
OS, % (95% CI)	64 (56–72)	68 (62–75)	61 (58–65)	0.1
GRFS, % (95% CI)	45 (37–53)	42 (35–50)	46 (42–50)	0.9

*Acute GvHD: 100-day cumulative incidence; cGvHD, NRM, and RI: cumulative incidence at 2 years; DFS, OS, and GRFS: survival probability at 2 years

*GvHD* graft-versus-host disease, *CI* confidence interval, *NRM* non-relapse mortality, *RI* relapse incidence, *LFS* leukemia-free survival, *OS* overall survival, *GRFS* graft-versus-host disease and relapse-free survival

**Table 3:** Multivariable analysis of transplants outcomes according to donor type

	MSD	MUD			Haplo		
Outcome	Reference	HR	95% CI	*p*	HR	95% CI	*p*
Acute GvHD							
Grades II–IV	1	1.42	0.92–2.19	0.11	**1.6**	**1.08–2.37**	**0.02**
Grades III–IV	1	1.22	0.56–2.64	0.61	1.76	0.92-3.37	0.09
Chronic GvHD							
Overall	1	0.97	0.64–1.46	0.88	1.22	0.84–1.76	0.92
Extensive type	1	0.98	0.57–1.7	0.95	0.87	0.53–1.42	0.57
NRM	1	1.38	0.73–2.6	0.3	**2.6**	**1.5–4.49**	**< 0.001**
RI	1	0.8	0.54–1.17	0.24	**0.67**	**0.48–0.93**	**0.02**
LFS	1	0.93	0.68–1.29	0.67	1.07	0.82-1.4	0.6
OS	1	0.91	0.64–1.31	0.62	1.17	0.86–1.59	0.32
GRFS	1	1.01	0.76–1.34	0.94	1.03	0.82–1.31	0.8

*GvHD* graft-versus-host disease, *CI* confidence interval, *NRM* non-relapse mortality, *RI* relapse incidence, *LFS* leukemia-free survival, *OS* overall survival, *GRFS* graft-versus-host disease and relapse-free survival

**Table 4:** Multivariable analysis of transplants outcomes

Variable	aGvHD II-IV		aGvHD III-IV		cGvHD		Ext. cGvHD		Relapse		NRM		LFS		OS		GRFS	
	HR (95% CI)	*p*	HR (95% CI)	*p*	HR (95% CI)	*p*	HR (95% CI)	*p*	HR (95% CI)	*p*	HR (95% CI)	*p*	HR (95% CI)	*p*	HR (95% CI)	*p*	HR (95% CI)	*p*
Donor type																		
MSD	1		1		1		1		1		1		1		1		1	
MUD	1.39 (0.9–2.15)	0.14	1.22 (0.56–2.64)	0.61	0.96 (0.64–1.45)	0.84	0.98 (0.57–1.7)	0.95	0.79 (0.54–1.16)	0.22	1.38 (0.74–2.61)	0.32	0.93 (0.68–1.29)	0.67	0.91 (0.63–1.3)	0.59	1.01 (0.76–1.33)	0.97
Haplo	**1.60** **(1.08**–**2.37)**	**0.02**	1.76 (0.92–3.37)	0.09	1.21 (0.84–1.75)	0.3	0.87 (0.53–1.42)	0.57	**0.67** **(0.48**–**0.93)**	**0.02**	**2.60** **(1.5**–**4.49)**	**< 0.001**	1.07 (0.82–1.4)	0.6	1.17 (0.86–1.6)	0.31	1.03 (0.81–1.3)	0.8
Patient’s age per 10 years^1^	1.05 (0.96–1.17)	0.29	1.00 (0.85–1.18)	0.97	1.03 (0.93–1.14)	0.57	0.95 (0.83–1.09)	0.48	0.97 (0.88–1.06)	0.49	**1.35** **(1.19**–**1.54)**	**< 0.001**	**1.09** **(1.02**–**1.18)**	**0.02**	**1.21** **(1.11**–**1.32)**	**< 0.001**	1.04 (0.97–1.11)	0.26
Karnofsky performance status																		
Poor (< 90)	1		1		1		1		1		1		1		1		1	
Good (≥ 90)	0.92 (0.68–1.24)	0.57	0.76 (0.47–1.23)	0.27	0.79 (0.59–1.07)	0.13	0.84 (0.55–1.3)	0.44	0.88 (0.66–1.19)	0.42	0.74 (0.53–1.04)	0.08	**0.8** **(0.65**–**0.99)**	**0.04**	0.83 (0.66–1.06)	0.13	0.8 (0.66–0.97)	0.03
Patient´s CMV serostatus																		
Negative	1		1		1		1		1		1		1		1		1	
Positive	0.97 (0.72–1.31)	0.86	1.21 (0.71–2.05)	0.49	0.86 (0.65–1.14)	0.28	**0.62** **(0.42**–**0.92)**	**0.02**	1.24 (0.92–1.68)	0.16	1.35 (0.91–1.99)	0.13	**1.27** **(1**–**1.6)**	**0.04**	1.24 (0.96–1.6)	0.1	1.02 (0.83–1.24)	0.87
Cytogenetic risk category																		
Poor	1		1		1		1		1		1		1		1		1	
Standard or intermediate	0.9 (0.66–1.22)	0.48	1.01 (0.59–1.7)	0.98	1.08 (0.79–1.48)	0.63	0.78 (0.5–1.21)	0.26	**0.58** **(0.43**–**0.78)**	**<0.001**	1.01 (0.69–1.48)	0.96	**0.73** **(0.59**–**0.92)**	**0.007**	0.7 (0.54–0.9)	0.005	0.78 (0.63–0.96)	0.02
Not available	0.77 (0.53–1.12)	0.17	0.73 (0.39–1.36)	0.32	0.92 (0.64–1.34)	0.66	0.65 (0.39–1.1)	0.11	**0.69** **(0.49**–**0.97)**	**0.03**	1 (0.65–1.54)	1	**0.79** **(0.61**–**1.02)**	**0.07**	0.81 (0.61–1.07)	0.14	0.81 (0.64–1.03)	0.08
Type of AML																		
De novo	1		1		1		1		1		1		1		1		1	
Secondary	0.86 (0.61–1.22)	0.4	1.03 (0.59–1.79)	0.92	1 (0.72–1.37)	0.98	0.83 (0.49–1.4)	0.48	1.20 (0.87–1.64)	0.27	1.08 (0.76–1.55)	0.67	1.18 (0.93–1.49)	0.17	1.20 (0.93–1.54)	0.16	1.11 (0.89–1.38)	0.36
Time from diagnosis to SCT^1,2^	0.93 (0.83–1.05)	0.24	0.85 (0.68–1.06)	0.16	0.86 (0.75–0.98)	0.03	0.9 (0.75–1.09)	0.29	0.93 (0.82–1.06)	0.29	1.02 (0.89–1.16)	0.79	0.98 (0.89–1.07)	0.6	1 (0.9–1.1)	0.93	0.96 (0.89–1.04)	0.37
Conditioning intensity																		
RIC	1		1		1		1		1		1		1		1		1	
MAC	1.01 (0.76–1.35)	0.93	1.12 (0.7–1.8)	0.64	1.04 (0.77–1.41)	0.81	0.75 (0.5–1.13)	0.17	**0.58** **(0.43**–**0.77)**	**<0.001**	0.95 (0.68–1.32)	0.74	**0.71** **(0.58**–**0.88)**	**0.001**	**0.76** **(0.60**–**0.96)**	**0.02**	**0.79** **(0.66**–**0.95)**	**0.01**
Stem cell source																		
Bone marrow	1		1		1		1		1		1		1		1		1	
Peripheral blood	**1.93** **(1.39**–**2.67)**	**< 0.001**	**1.86** **(1.1**–**3.15)**	**0.02**	**1.72** **(1.24**–**2.39)**	**0.001**	**1.86** **(1.15**–**3.01)**	**0.01**	**0.74** **(0.55**–**0.99)**	**0.04**	1.07 (0.76–1.51)	0.69	0.9 (0.73–1.11)	0.31	0.96 (0.75–1.22)	0.73	1.13 (0.93–1.38)	0.2
Donor-recipient gender																		
Other combinations	1		1		1		1		1		1		1		1		1	
Female donor to male recipient	0.87 (0.63–1.18)	0.37	1.05 (0.63–1.75)	0.85	1.19 (0.9–1.58)	0.23	**1.71** **(1.14**–**2.57)**	**0.009**	0.87 (0.64–1.2)	0.4	1.11 (0.79–1.56)	0.54	0.99 (0.79–1.24)	0.94	1.00 (0.79–1.29)	0.97	1.08 (0.88–1.32)	0.48
Center effect		0.04		0.27		0.01		0.28		0.22		0.06		0.25		0.13		0.28

^1^Continuous variable

^2^By 2 months increase

The cumulative incidence of cGvHD at 2 years was 30% (95% CI; 26–33), 32% (95% CI; 25–39), and 34% (95% CI; 26–41) (*p* = 0.3) for Haplo, MUD, and MSD, respectively (Table 2). The cumulative incidence of extensive type cGvHD was 10% (95% CI; 8–13) for Haplo, 18% (95% CI; 13–25) for MUD, and 14% (95% CI; 9–20) for MSD (*p* = 0.003) (Table 2). In multivariable analysis (Table 4), use of PB was independently associated with a higher risk of cGvHD (HR 1.71; 95% CI, 1.23–2.39; *p* = 0.001) and extensive cGvHD (HR 1.86; 95% CI, 1.15–3.01; *p* = 0.01). Female donor to male recipient showed also an increased risk of extensive cGvHD (HR 1.71; 95% CI, 1.14–2.57; *p* = 0.009). Donor type was not associated with the risk of cGvHD or extensive cGvHD in multivariate analysis.

### Relapse

**Fig. 1:**
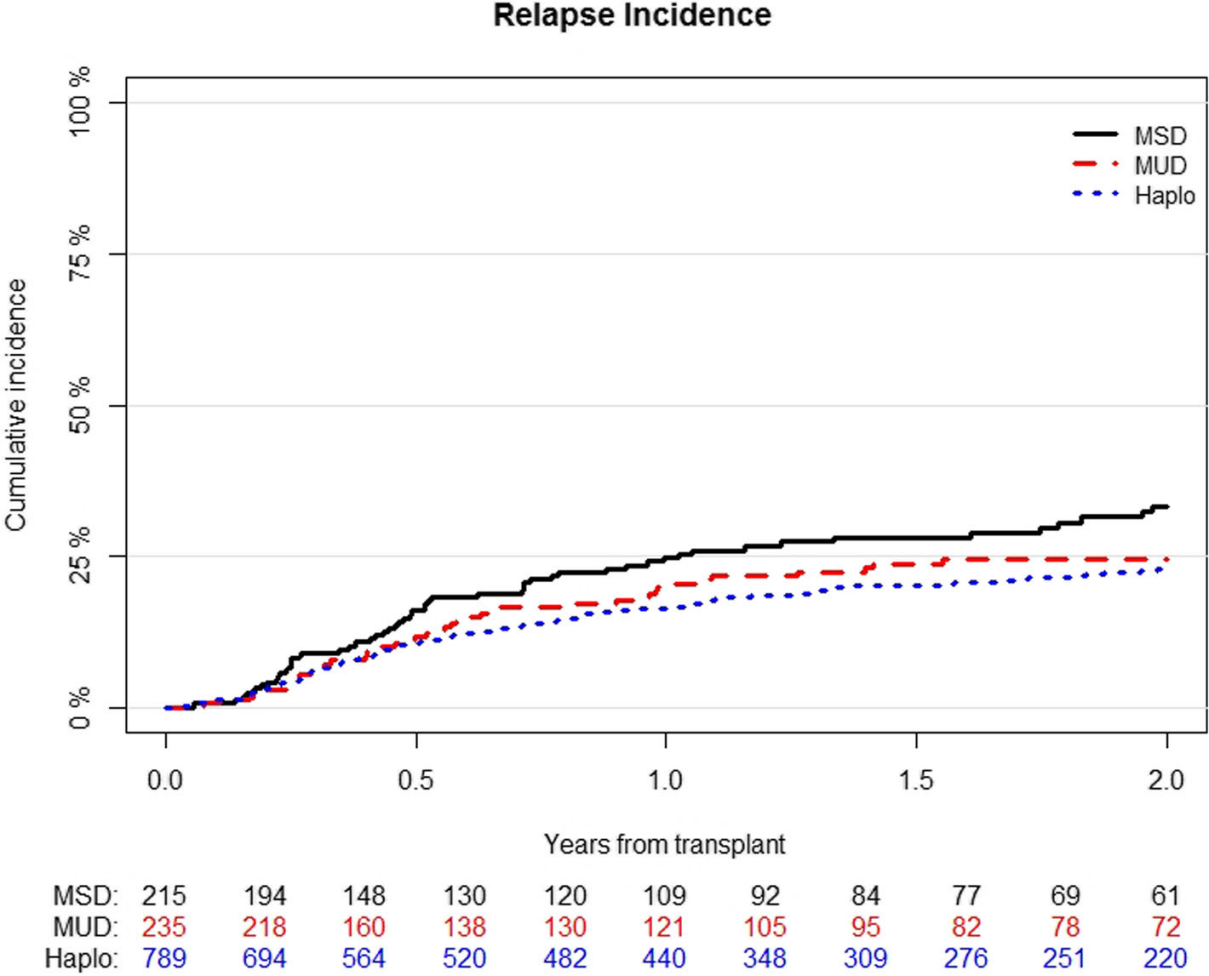
Cumulative incidence of relapse according to the type of transplant

The median time to relapse was 192 days (range, 3–1750). The cumulative incidence of relapse at 2 years was 23% (95% CI; 20–26) for Haplo, 25% (95% CI; 19–31) for MUD, and 33% (95% CI; 26–41) for MSD (*p* = 0.02) (Table 2) (Fig. 1). In multivariable analysis (Table 3), Haplo was associated with a lower risk of relapse when compared with MSD (HR 0.67; 95% CI, 0.48–0.93; *p* = 0.02). Other variables associated with a decreased risk of relapse were use of MAC (HR 0.56; 95% CI, 0.43–0.77; *p* < 0.001), PB as the stem cell source (HR 0.74; 95% CI, 0.45–0.99; *p* = 0.04), and standard or intermediate cytogenetic risk category (HR 0.58; 95% CI, 0.43–0.78; *p* < 0.001) (Table 4).

### NRM and causes of death

**Fig. 2:**
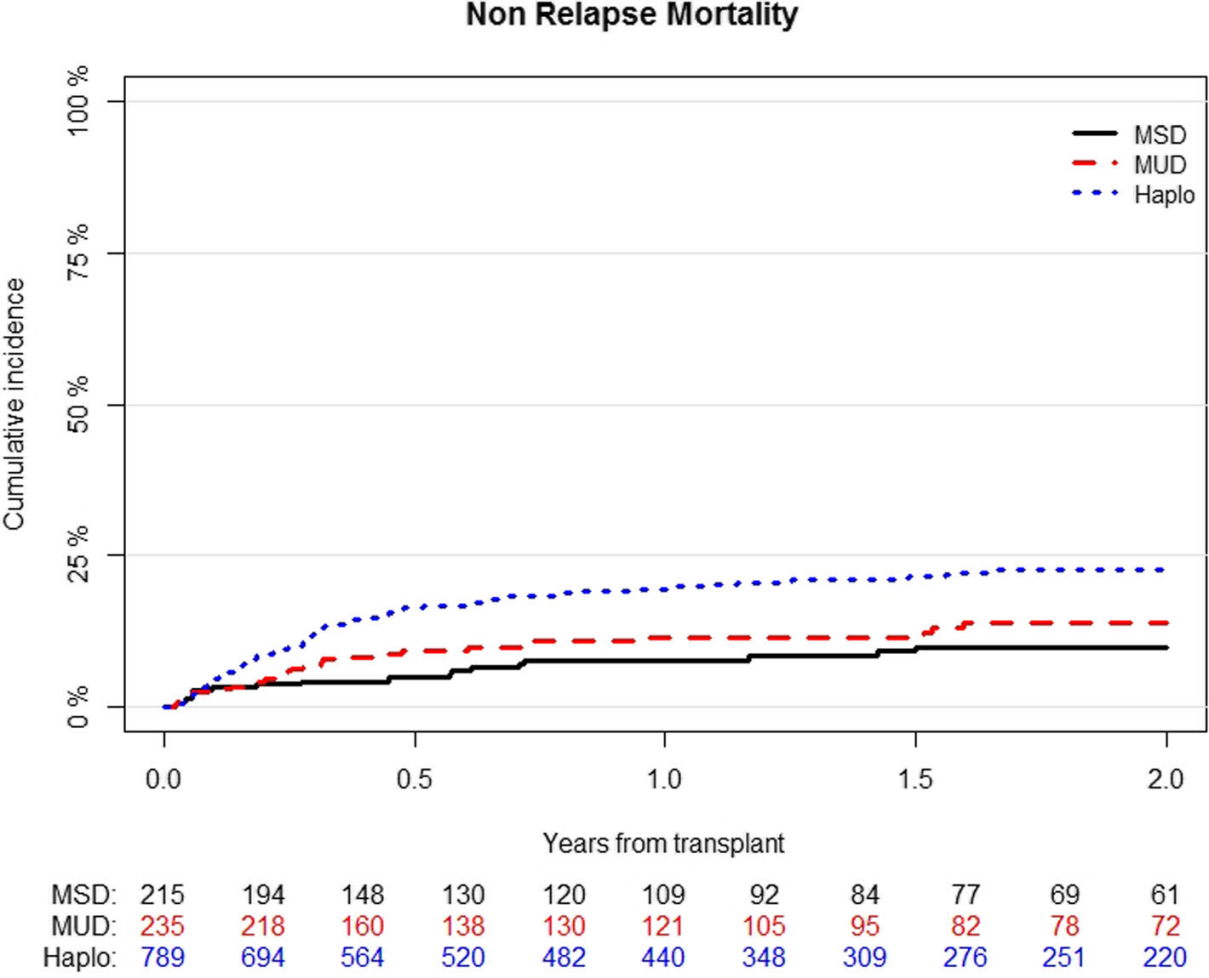
Cumulative incidence of non-relapse mortality according to the type of transplant

The cumulative incidence of NRM at 2 years was 23% (95% CI; 20–26) for Haplo, 14% (95% CI; 9–19) for MUD, and 10% (95% CI; 6–15) for MSD (*p* < 0.001) (Table 2) (Fig. 2). In multivariable analysis (Table 3), Haplo was associated with an increased risk of NRM (HR 2.6; 95% CI, 1.5–4.49; *p* < 0.001). The other factor associated with an increased NRM was higher recipient’s age per 10 years (HR 1.35; 95% CI, 1.19–1.54; *p* < 0.001) (Table 4).

At the last follow-up, 432 patients had died, of which 290 (65%) were due to a variety of non-relapse causes, 222 (73%) in Haplo, 36 (52%) in MUD, and 32 (47%) in MSD. The main causes of transplant-related deaths were infections and GvHD, being 107 (39%) and 40 (14%) in Haplo, 13 (20%) and 8 (12%) in MUD, and, 10 (15%) and 11 (16%) in MSD cohorts, respectively (Table 5).

**Table 5:** Causes of death according to donor type

Causes of death	MSD *n* (%)	MUD *n* (%)	Haplo *n* (%)
Relapse	36 (53)	31 (48)	75 (27)
Infections	10 (15)	13 (20)	107 (39)
GvHD	11 (16)	8 (12)	40 (14)
Interstitial pneumonitis	3 (4)	4 (6)	6 (2)
Sinusoidal obstruction syndrome	0 (0)	1 (2)	7 (3)
Hemorrhage	1 (1)	1 (1)	4 (1)
Secondary malignancy	0 (0)	0 (0)	6 (2)
Graft failure	0 (0)	0 (0)	5 (2)
Other	7 (10)	7 (10)	27 (10)

### Survival

For the entire cohort, LFS, OS, and GRFS at 2 years were 56% (95%CI; 53–59), 63% (95%CI; 60–66), and 45% (95%CI; 42–48), respectively.

**Fig. 3:**
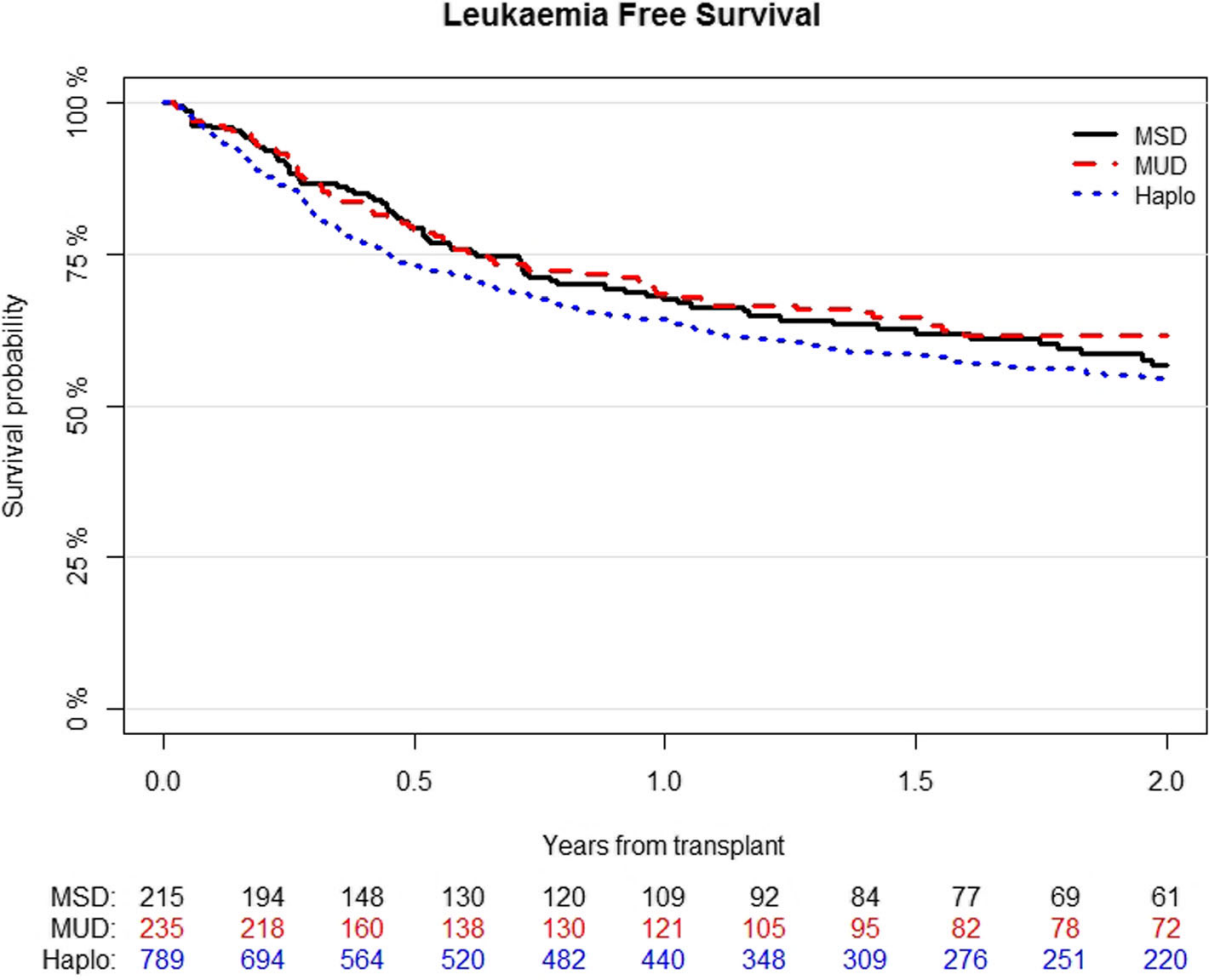
Probability of leukemia-free survival according to the type of transplant

LFS was 54% (95% CI; 51–58) for Haplo, 62% (95% CI; 55–69) for MUD, and 57% (95% CI; 49–65) for MSD (*p* = 0.2) (Fig. 3). In multivariable analysis (Table 4), variables associated with better LFS were MAC (HR 0.71; 95% CI, 0.58–0.88; *p* = 0.001), good- or intermediate-risk cytogenetics (HR 0.73; 95% CI, 0.58–0.92; *p* = 0.007), and good performance status (HR 0.8; 95% CI, 0.64–0.99; *p* = 0.04), while higher recipient’s age per 10 years (HR 1.1; 95% CI, 1.02–1.18; *p* = 0.02) and positive CMV serostatus of the recipient (HR 1.27; 95% CI, 1–1.6; *p* = 0.04) showed worse outcome.

**Fig. 4:**
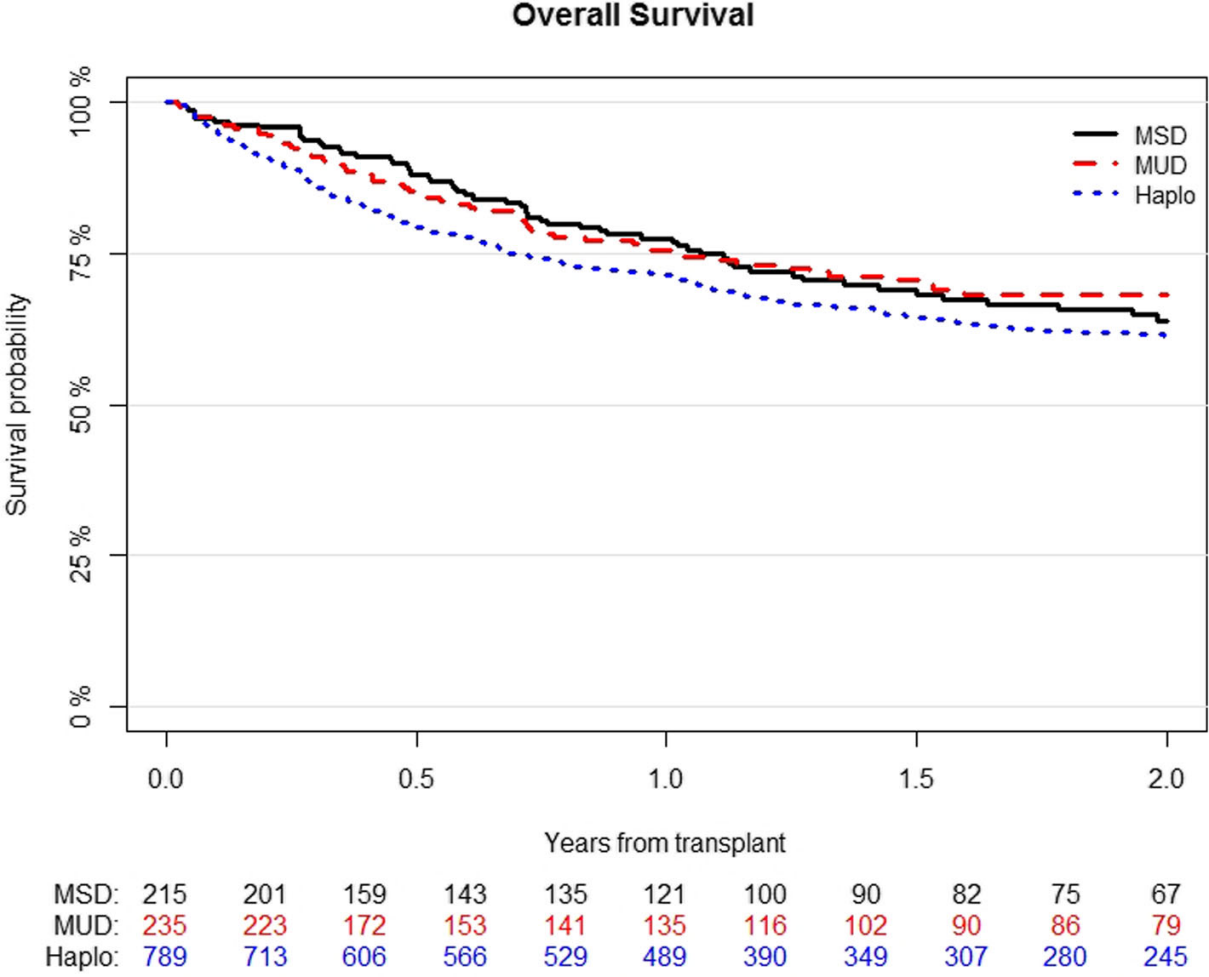
Probability of overall survival according to the type of transplant

OS was 61% (95% CI; 58–65) for Haplo, 68% (95% CI; 62–75) for MUD, and 64% (95% CI; 56–72) for MSD (*p* = 0.1) (Figure 4). Variable that independently correlated with better OS in multivariable analysis were MAC (HR 0.76; 95% CI, 0.6–0.96; *p* = 0.02) and good- or intermediate-risk cytogenetics (HR 0.7; 95% CI, 0.54–0.9; *p* = 0.005), while higher recipient’s age per 10 years was associated with poorer survival (HR 1.22; 95% CI, 1.12–1.33; *p* < 0.001) (Table 4).

GRFS was 46% (95% CI; 42–50) for Haplo, 42% (95% CI; 35–50) for MUD, and 45% (95% CI; 37–53) for MSD (*p* = 0.9) (Table 2). For GRFS, MAC (HR 0.79; 95% CI, 0.66–0.95; *p* = 0.01), good- or intermediate-risk cytogenetics (HR 0.78; 95% CI, 0.63–0.96; *p* = 0.02), and good performance status (HR 0.8; 95% CI, 0.67–0.97; *p* = 0.02) were associated with improved outcome (Table 4).

## Discussion

The use of PTCy for GvHD prophylaxis in patients with AML in CR1 receiving SCT from MSD, MUD, and Haplo is safe and effective, resulting in low rates of GVHD, especially chronic, in all transplant settings. Using this approach, our results demonstrate that patients undergoing Haplo-SCT had higher rates of aGVHD and NRM, but lower relapse incidence. As seen in other transplant scenarios, PB was also associated with more GVHD and less relapse.

Due to the retrospective nature of a registry-based study, some potential bias cannot be completely ruled out. In order to minimize one of the most important, such as the disease status at transplantation, all patients included in the analysis had AML in CR1. Although all patients received PTCy prophylaxis as the main inclusion criteria for the study, a variety of conditioning regimens were used and there were obvious differences in additional GvHD prevention strategies, such as in vivo TCD or the addition of other IS drugs, depending of the type of donor. In fact, Haplo patients received more frequently MAC, BM, and PTCy with 2 IS drugs, while a higher proportion of MUD patients received in vivo TCD. Although some of these variables could be adjusted in multivariable analysis, TCD and combination of IS drugs for GvHD prophylaxis were strongly associated with type of donor and their effect could not be evaluated. Despite all these pitfalls, we aimed to compare MSD, MUD, and Haplo using a homogeneous GvHD prophylaxis with PTCy in a large series of patients, which allowed us to segregate the effect of donor from the effect of GvHD prophylaxis.

PTCy was highly effective in preventing acute and chronic GvHD in MSD, MUD, and Haplo-SCT and seems to compare favorably with standard GvHD prophylaxis with calcineurin inhibitor and methotrexate in MSD and MUD transplants [24]. In fact, the incidence of GvHD after PTCy seems similar to that reported with anti-T cell globulin in both scenarios [25], with the potential advantage of avoiding complications associated with prolonged TCD. An interesting observation was the higher incidence of acute grades II–IV and a trend towards a higher severe acute GvHD of Haplo-SCT compared to MSD transplants. It should be noted that most previous studies had not been able to demonstrate this effect [9, 11, 14, 26], probably due to the use of different GvHD prophylaxis for each procedure. In fact, Haplo was associated with increased risk of GVHD compared to MSD in two prospective studies using similar non-PTCy transplant platforms [16, 17]. Particularly relevant was the low overall chronic and chronic extensive GvHD observed in our study in all cohorts, as it has been previously reported with PTCy [1, 4, 7, 24, 27]. We should highlight that we did not observe increased risk of chronic GvHD in Haplo in multivariable analysis. We could speculate that PTCy abrogates the detrimental effect of HLA disparity for this particular outcome, but we should also consider that most patients in the Haplo cohort received PTCy with a double combination of IS drugs, while patients in the MSD and MUD cohorts received less intensive GvHD prophylaxis. In fact, a recent study of the ALWP-EBMT has recently reported that the addition of IS drugs to PTCy enhances its effect and reduces the risk of severe chronic GvHD, reducing mortality and improving survival [6].

The most important observation from our study is that, using PTCy as GVHD prophylaxis, NRM was higher in the Haplo setting compared with the MSD and MUD cohorts. Previous studies comparing Haplo (with PTCy) with MSD and MUD transplants (with standard GvHD prophylaxis) have reported discrepant results in terms of NRM. While some studies reported a higher rate of NRM in Haplo [11, 15], some others reported similar [12, 14] or even improved outcomes [10]. The reasons of these discrepancies remain unexplained and are probably multifactorial, but differences in transplant platforms could explain, at least in part, some of these results. Under similar GvHD prophylaxis, a greater HLA disparity in the Haplo compared with the MSD and MUD settings could explain a higher NRM. This finding suggests that HLA-matched donors, when available, should remain as the first choice. Although the negative impact of Haplo in NRM was partially counterbalanced with a decreased incidence of relapse that translated in similar LFS, other strategies aiming at reducing relapse such as maintenance or MRD-guided therapy with a growing targeted therapy strategies could be investigated.

The fact that Haplo was associated with lower risk of relapse deserves special attention. It is possible that this was a spurious finding since more patients in the Haplo cohort died from NRM and were therefore no longer at risk of relapse. However, Haplo procedure could have offered enhanced anti-leukemic efficacy, intriguingly in a way that was independent of chronic GVHD. Superior graft-versus-leukemia effect of Haplo compared to MSD transplants for high-risk AML has already been observed in previous comparative studies. Two prospective trials with biological randomization from China showed decreased posttransplant minimal residual disease (MRD) positivity [28] or relapse [29] in patients undergoing Haplo, particularly relevant for those with detectable pretransplant MRD. In addition, a recent retrospective study of EBMT also showed decreased relapse incidence in patients with high-risk cytogenetics undergoing Haplo [15].

The immunological pressure of Haplo grafts has been illustrated with the observation that loss of the mismatched HLA haplotype is a frequent mechanism of escape associated with relapse [30]. The biological explanation is unknown but NK-mediated alloreactivity has been previously proposed to induce enhanced efficacy and GVHD protection in the context of T cell-depleted Haplo-SCT [31]. The hypothesis of an increased anti-leukemic efficacy independent of GvHD of Haplo-SCT compared to matched donors in the context of PTCy should be further explored from a clinical and biological point of view.

Despite the risks and benefits of BM over PB have been widely investigated, the effect of the stem cell source on transplant outcomes deserves special consideration. In MUD transplants, BM reduced the risk of chronic GVHD in a randomized study [32] and improved long-term GRFS and overall survival in a large retrospective registry study [33]. In Haplo-SCT, the use of PB resulted in an increased risk of acute GvHD, uncertain impact of chronic GvHD, and decreased risk of relapse in patients with acute leukemia, but not with lymphoma [34, 35]. In the present study, we confirm that PB was associated with increased risk of acute and chronic GvHD, less relapses, but no final influence on NRM and survival.

Although most retrospective studies comparing MAC with RIC in patients with AML have suggested similar survival, since the latter has been associated with increased relapse but reduced NRM [36, 37], we observed a significant reduction of relapse with MAC that translated into improved survival when compared with RIC. Unfortunately, the only randomized study, designed to address this issue in patients with AML, was closed to patient accrual early due to excess of relapse and reduced survival in the RIC cohort [38]. Since the efficacy of RIC in SCT mainly relies on graft-versus-leukemia effect, it may be particularly relevant to increase conditioning intensity in transplant platforms with effective GvHD control such as with the use of PTCy.

## Conclusion

In patients with AML undergoing allo-SCT, PTCy for GvHD prophylaxis showed promising outcomes. Future studies comparing PTCy standard regimens are warranted to establish the standard of care. In this specific scenario, Haplo-SCT had increased risk of acute GVHD and NRM that was counterbalanced by a lower relapse incidence that translated into no significant difference in LFS and OS. Haplo-SCT offers a good alternative to matched donor transplants.

## Data Availability

The dataset supporting the conclusions of this article are available in the ALWP of EBMT in Paris, Saint Antoine Hospital.

## Abbreviations

aGVHD: Acute GVHD
allo-SCT: Allogeneic stem cell transplantation
ALWP: Acute Leukaemia Working Party
AML: Acute myeloid leukemia
BM: Bone marrow
cGVHD: Chronic GVHD
CR1: First complete remission
EBMT: European Society for Blood and Marrow Transplantation
GRFS: GVHD-free and relapse-free survival
GVHD: Graft-versus-host
Haplo: Haploidentical donors
HLA: Human leukocyte antigen
LFS: Leukemia-free survival
MAC: Myeloablative conditioning
MSD: Matched sibling donors
MUD: Matched unrelated
NRM: Nonrelapse mortality
PB: Peripheral blood
PTCy: Post-transplant cyclophosphamide
RIC: Reduced intensity conditioning
SCT: Stem cell transplantation
OS: Overall survival
